# Effects of Optimized Key Processing Parameters on Macro-Compositions and Volatile Organic Compounds in High-Altitude Oolong Tea (*Camellia sinensis* (L.) O. Kuntze)

**DOI:** 10.3390/plants15142125

**Published:** 2026-07-09

**Authors:** Wenjing Zhang, Yuting Li, Shuaibo Shao, Xingyuan Yao, Chengzhe Zhou, Zhong Wang, Yuqiong Guo

**Affiliations:** 1 Anxi College of Tea Science, Fujian Agriculture and Forestry University, Anxi County, Quanzhou 362400, China; 2 Fujian Provincial Crop Production Technology Extension Station, Fujian Provincial Department of Agriculture and Rural Affairs, Fuzhou 350003, China; 3 Fujian Collaborative Innovation Center for Green Cultivation and Processing of Tea Tree in Universities, Fujian Agriculture and Forestry University, Anxi County, Quanzhou 362400, China

**Keywords:** high-altitude Oolong Tea, processing parameter optimization, tea quality, multivariate statistical analysis

## Abstract

High-altitude environments provide a favorable basis for tea quality formation, but suitable processing parameters for high-altitude Oolong Tea (HAOT, *Camellia sinensis* (L.) O. Kuntze) remain unclear. This study optimized key processing parameters for HAOT using a three-factor, three-level orthogonal design involving withering method, shaking intensity and standing time. Sensory evaluation, quantitative descriptive analysis (QDA), macro-compositions determination, headspace solid-phase microextraction coupled with gas chromatography–mass spectrometry (HS-SPME-GC-MS), multivariate statistics and correlation analysis were used to clarify quality differences and their chemical basis. Leaf anatomical observation showed that high-altitude fresh leaves had a thicker cuticle and more compact mesophyll structure, suggesting the need for targeted processing regulation. Range analysis indicated that shaking intensity had the largest R value for sensory scores, followed by withering method, whereas standing time showed a comparatively small R value. The favorable combination was combined withering, heavy shaking (6 rounds) and 60 min standing, hereafter referred to as FHM. FHM showed the highest floral and fruity aroma scores and produced a mellow, full-bodied taste with a sweet aftertaste. It also contained significantly higher flavonoid and soluble sugar levels than the other processing combinations. A total of 65 volatile organic compounds were detected, with terpenoids and esters as the dominant classes. ROAV analysis identified geraniol, linalool, β-ionone, (*E*)-nerolidol and benzaldehyde as representative major aroma-active compounds, while geraniol and linalool were especially prominent in FHM. Correlation analysis further linked flavonoids and soluble sugars with positive taste attributes, and linalool and geraniol with floral–fruity aroma. These results provide a practical basis for optimizing HAOT processing and improving product quality.

## 1. Introduction

The traditional view that “high mountains and mist produce good tea” reflects the long-recognized influence of mountain environments on tea quality. As a perennial evergreen crop, tea plants (*Camellia sinensis* (L.) O. Kuntze) are adapted to warm and humid environments and moderate light or partial shading conditions. High-altitude tea gardens are characterized by large day–night temperature differences, high humidity and abundant diffuse light, all of which are key ecological factors shaping high-quality tea [1]. Previous studies have shown that the special ecological conditions of high-altitude environments can significantly affect the accumulation and transformation of secondary metabolites in tea plants, thereby giving high-mountain tea a fresher, softer taste and a more pronounced, elegant aroma [2]. Current research on the effects of altitude on tea quality has mainly focused on green tea and white tea. For example, terpenes and aromatic alcohols were significantly increased in high-altitude white tea, producing a more delicate floral aroma [3], while amino acids and other taste-related compounds accumulate more strongly in high-altitude green tea, improving freshness and sweetness [4]. Previous work has suggested a possible difference in leaf structure between high- and low-altitude tea plants [5], but direct evidence linking such structural characteristics to HAOT processing remains limited. Therefore, in this study, leaf anatomical observation was first conducted to provide a structural basis for the subsequent optimization of processing parameters.

Oolong Tea is a representative semi-fermented tea. Its rich floral–fruity aroma and mellow, sweet aftertaste are shaped jointly by cultivar, ecological environment and processing technique [6]. Oolong Tea processing mainly includes withering, green-making (including shaking and standing), fixation, rolling and drying. Among these steps, withering and green-making are critical for the formation of the distinctive floral–fruity flavor of Oolong Tea [7]. Withering can be performed in several ways, including solar withering, indoor withering and combined withering, and different withering methods have distinct effects on tea quality [8]. Solar withering exposes fresh leaves to natural light and heat, leading to relatively rapid water loss and physiological responses, whereas indoor withering provides milder conditions and allows a slower dehydration process [9,10]. These differences may influence enzymatic activity and metabolite transformation during withering, thereby affecting the subsequent formation of tea aroma and taste quality. Combined withering is commonly used in white tea processing and can significantly improve aroma and taste quality [11].

Shaking is also a key step in Oolong Tea quality formation and strongly affects metabolite transformation and accumulation in tea leaves [12]. Previous studies have shown that low-altitude fresh leaves subjected to light shaking produce a fresh infusion, with relatively high levels of indole, α-farnesene, jasmine lactone and phenylethyl alcohol among volatile organic compounds, resulting in a noticeable floral–fruity aroma [13]. Heavy shaking, in contrast, can produce a richer and sweeter taste and a more complex aroma dominated by terpene alcohols such as nerolidol, geraniol and benzyl alcohol [14]. Standing after shaking is an important buffering stage during green-making. It regulates water redistribution and metabolism and has a strong effect on final tea quality [15]. Standing time has also been reported to be particularly important for the formation of the characteristic quality of high-catechin Oolong Tea [16]. When shaking and standing are incorporated into black tea processing, sweetness and floral aroma can be improved, whereas bitterness and astringency are reduced [17,18]. These findings indicate that withering method, shaking intensity and standing time affect tea taste and aroma to different extents. Scientific selection and regulation of processing parameters are therefore essential for producing high-quality high-altitude Oolong Tea (HAOT). However, the relative effects of the withering method, shaking intensity and standing time on the quality formation of HAOT remain unclear.

In this study, fresh leaves from a high-altitude tea garden were used as raw material. A three-factor, three-level orthogonal experiment was designed around the withering method, shaking intensity and standing time to systematically compare the effects of different processing combinations on Oolong Tea quality. Sensory evaluation, conventional biochemical analysis and HS-SPME-GC-MS were used to compare biochemical components and volatile organic compounds among processing parameters and to identify key aroma components responsible for aroma differences. The results provide a theoretical basis for optimizing the processing of HAOT.

## 2. Results and Discussion

### 2.1. Comparison of Leaf Anatomical Structures Between High- and Low-Altitude Tea Plants

**Figure 1:**
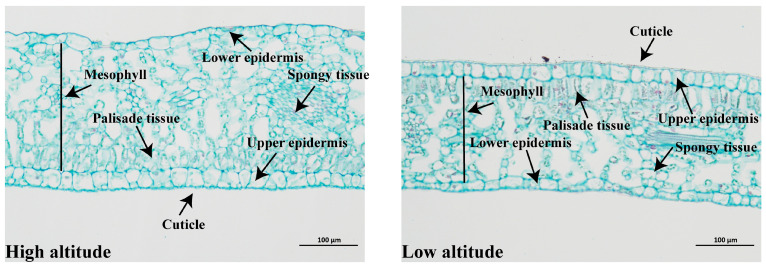
Comparative leaf anatomy of tea plants at high and low altitudes.

High-altitude tea gardens are generally exposed to lower air temperatures and stronger solar radiation, and their soil physicochemical properties and microbial communities also differ from those of low-altitude gardens. These factors together can promote adaptive changes in leaf anatomical structure. As shown in Figure 1, the overall leaf thickness of high-altitude tea plants was clearly greater than that of low-altitude samples. The spongy tissue of high-altitude fresh leaves was more compact, whereas the spongy tissue of low-altitude leaves was looser and contained larger intercellular spaces. The cuticle of high-altitude leaves was also thicker. Previous studies have shown that the thickness of tea leaves and their tissues, including epidermis, cuticle, palisade tissue, and spongy tissue, generally increases with altitude, resulting in a more compact mesophyll structure [19]. These structural characteristics may influence key steps during Oolong Tea processing. For example, the cuticle is an important barrier limiting water loss from leaves, while withering is closely associated with dehydration, leaf softening, and subsequent biochemical transformation [20]. Therefore, the thicker cuticle of high-altitude fresh leaves may affect water loss behavior during withering and may lead to differences in the rate and uniformity of dehydration under different withering methods [21]. In addition, the compact mesophyll structure may affect the degree of cell disruption caused by mechanical force. Since shaking is a critical step in Oolong Tea processing that induces moderate mechanical damage, increases cell membrane permeability, and promotes contact between enzymes and substrates [22], shaking intensity was selected as an important processing factor. Meanwhile, the standing time after shaking was also taken into consideration, as it can regulate water redistribution and provide time for further transformation of endogenous compounds during the green-making process [23]. Based on these considerations, the withering method, shaking intensity, and standing time were selected as three representative processing factors for the orthogonal optimization of HAOT.

### 2.2. FHM Identified as the Favorable Processing Combination

The factor and level settings for the orthogonal experiment are shown in Table S1. Three processing factors were considered, including the withering method (A), shaking intensity (B), and standing time (C), each of which was set at three levels. The levels of these factors were determined based on the characteristics of Oolong Tea processing, preliminary trials, relevant literature [14,17,24], and practical processing feasibility. For the withering method, solar withering, indoor withering, and combined withering were selected because they represent the major withering methods used in Oolong Tea manufacture. Solar withering provides natural light and heat stimulation but is strongly affected by weather conditions, whereas indoor withering offers milder and more controllable conditions. Combined withering was therefore included to integrate the effects of outdoor solar exposure and indoor dehydration. Shaking intensity was mainly regulated by changing the number of shaking rounds, because the number of rounds is a practical and controllable variable for distinguishing light, moderate, and heavy shaking under production conditions. The number of shaking rounds also needs to be adjusted according to cultivar, fresh leaf status, and withering degree. Therefore, based on preliminary trials and practical processing feasibility, the number of shaking rounds was used as the basis for intensity classification, with four, five, and six rounds representing light, moderate, and heavy shaking, respectively. For standing time, considering that fresh leaves require a certain period after shaking for water redistribution and metabolite transformation, 60 min was selected as the moderate level. On this basis, 40 and 80 min were set by extending 20 min below and above this level to represent relatively short and long standing intervals, respectively. The orthogonal test results (Table 1) showed that shaking intensity had the largest R value, followed by the withering method and standing time. Based on the k values, the favorable processing combination was A3B3C2, corresponding to FHM, which consisted of combined withering, heavy shaking, and 60 min standing. This parameter was therefore selected as the recommended processing parameter for further analysis. In addition, the sensory quality of FHM met the special-grade standard for Rougui Oolong Tea according to GB/T 30357.5-2015 (Table S2) [25], further supporting its suitability as the optimal processing parameter. Based on these results, six representative processing combinations were selected for biochemical and volatile organic compound analyses: FHM (combined withering, heavy shaking, 60 min standing), FMS (combined withering, moderate shaking, 40 min standing), RHL (solar withering, heavy shaking, 80 min standing), RMM (solar withering, moderate shaking, 60 min standing), NML (indoor withering, moderate shaking, 80 min standing), and NHS (indoor withering, heavy shaking, 40 min standing). Tea samples subjected to light shaking (RLS, NLM and FLL) generally received low sensory scores and retained noticeable green notes, which did not conform well to the typical quality characteristics expected for Oolong Tea. Therefore, they were not included in subsequent component analysis.

### 2.3. Different Processing Parameters Resulted in Distinct Sensory Profiles

**Figure 2:**
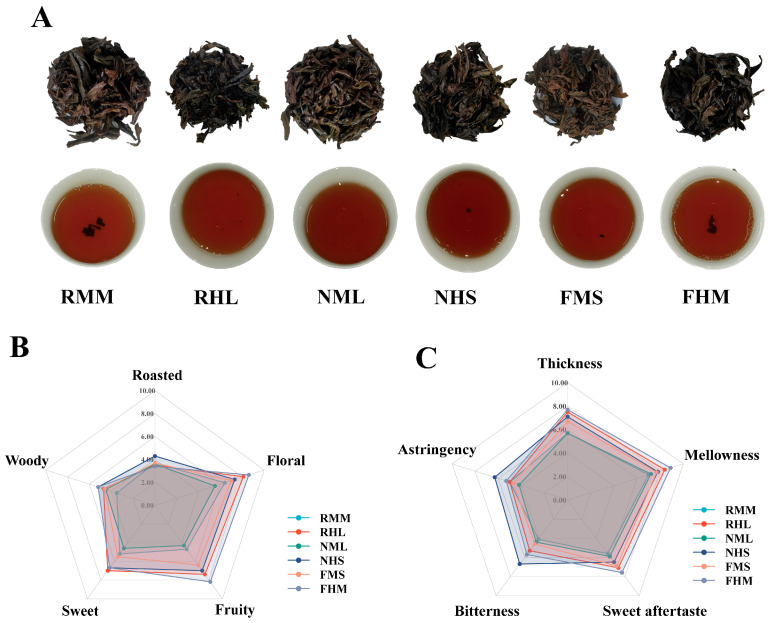
Sensory characteristics of HAOT. (**A**) Sensory evaluation of HAOT. (**B**) Aroma profiles based on QDA. (**C**) Taste profiles based on QDA.

The representative images of tea infusions and infused leaves are shown in Figure 2A, while the detailed sensory evaluation results are presented in Table S2. The quantitative descriptive analysis (QDA) results further indicated that different processing parameters exerted marked effects on the sensory characteristics of HAOT (Figure 2B,C and Figure S1). In terms of aroma attributes, FHM showed the highest scores for floral and fruity aromas, with values of 8.6 and 8.2, respectively, both of which were significantly higher than those of the other parameters. This result suggested that FHM produced a prominent floral–fruity aroma profile. RHL also exhibited relatively strong floral, fruity, and sweet aroma intensities, whereas RMM and NML showed comparatively lower overall aroma intensity. In contrast, the roasted aroma scores varied only slightly among the different parameters, indicating that the differences in aroma quality were mainly reflected in floral, fruity, sweet, and woody aroma attributes rather than roasted notes. For taste attributes, FHM had the highest scores for mellowness and sweet aftertaste, with values of 8.9 and 7.6, respectively, and also showed a relatively high thickness score. These results indicated that FHM produced a more mellow, full-bodied taste with a pronounced sweet aftertaste. RHL similarly showed relatively high scores for thickness and mellowness, whereas RMM and NML had lower scores for thickness and sweet aftertaste. In comparison, NHS had the highest bitterness and astringency scores, suggesting that its taste profile was less balanced. Overall, FHM exhibited a relatively balanced sensory profile, with advantages in both floral–fruity aroma and taste-related attributes. This result was consistent with the orthogonal test results, which identified FHM as a recommended processing parameter.

### 2.4. Processing Parameters Altered Quality-Related Macro-Compositions

**Figure 3:**
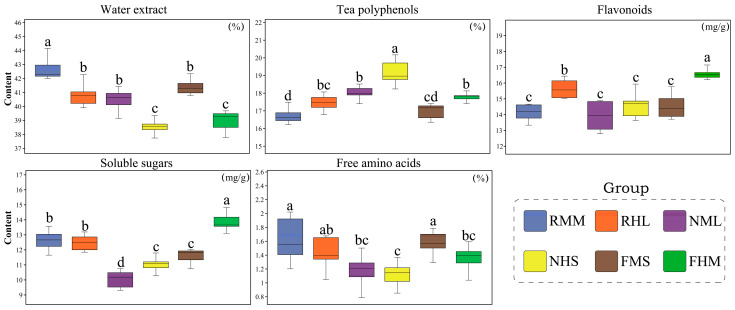
Biochemical component contents of HAOT under different processing techniques. Different letters indicate significant differences according to Duncan’s test (*p* < 0.05).

The effects of different processing techniques on biochemical components in Oolong Tea are shown in Figure 3. Tea polyphenol content was highest in NHS (19.07%), significantly higher than in the other parameters (*p* < 0.05), whereas RMM had the lowest tea polyphenol content (16.62%). FHM had the highest flavonoid and soluble sugar contents, at 16.48 mg/g and 13.71 mg/g, respectively, both significantly higher than those of the other parameters (*p* < 0.05). This result may be partly related to the stronger mechanical stimulation caused by the six-round shaking treatment in FHM. Considering the thicker cuticle and more compact mesophyll structure of high-altitude fresh leaves, stronger shaking may have enhanced tissue disruption and subsequent biochemical transformation, which was reflected in the higher flavonoid and soluble sugar contents observed in FHM. Previous studies have shown that soluble sugars are major contributors to sweetness and thickness in tea infusion and are significantly positively correlated with sweetness scores and overall sensory scores [26,27]. Flavonoids and their oxidation products are key compounds related to bitterness, astringency, and convergence of tea infusion; within an appropriate range, they can enhance mouthfeel thickness and enrich aroma characteristics. Therefore, the higher levels of flavonoids and soluble sugars in FHM may provide an important material basis for its improved sensory quality [28].

Water extract and free amino acids showed broadly similar trends across processing parameters. Overall, processing combination affected all measured biochemical components. Shaking intensity was an important factor because shaking mechanically damages leaf edge tissues and thereby affects tea quality. Previous studies have reported that heavier shaking may enhance mechanical stress and enzymatic oxidation, which may contribute to changes in liquor color and taste quality [16]. Thus, in the sensory evaluation, RHL, NHS, and FHM produced infusions with a more mellow taste and deeper liquor color, consistent with the findings of Wang et al. [13]. Compared with heavy-shaking parameters, moderate-shaking parameters showed relatively higher water extract and free amino acid contents, which may be associated with differences in mechanical stress and biochemical transformation.

### 2.5. Processing Parameters Affected the Composition and Distribution of Volatile Organic Compounds in HAOT

**Figure 4:**
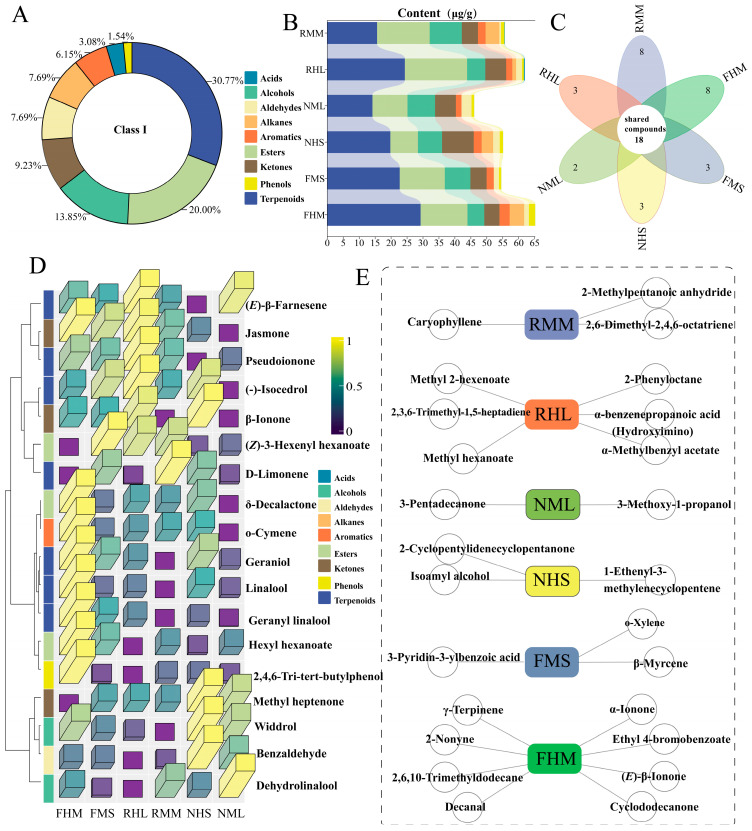
Class distribution of volatile organic compounds and analysis of key characteristic compounds in samples subjected to different parameters. (**A**) Class composition and percentage of volatile organic compounds; (**B**) relative contents of volatile organic compound classes under different processing conditions; (**C**) Venn diagram of shared and parameter-specific volatile organic compounds in Oolong Tea under different parameters; (**D**) heatmap of relative contents of shared volatile organic compounds; (**E**) volatile organic compounds specific to each parameter.

HS-SPME-GC-MS was used to determine volatile organic compounds in tea samples produced under different processing conditions. A total of 65 volatile organic compounds were detected, including 20 terpenoids, 13 esters, 9 alcohols, 6 ketones, 5 aldehydes, 5 alkanes, 4 aromatics, 2 acids, and 1 phenol. Their relative contents differed markedly among parameters. As shown in Figure 4A, terpenoids and esters were the main aroma compound classes and together accounted for more than 50% of the total volatile organic compounds in different tea samples. Terpenoids are important aromatic compounds in tea and are usually associated with floral, fruity, and woody notes [29]. In this study, the relative contents of terpenoid were significantly higher in RHL and FHM, reaching 24.32% and 29.26%, respectively, than in the moderate-shaking parameters (RMM, 15.62%; NML, 14.21%). Esters, which are associated with sweet and floral notes [30,31], were relatively abundant in sun-withering parameters (RMM, 16.47%; RHL, 19.51%) and combined-withering parameters (FMS, 14.13%; FHM, 14.68%), but were significantly lower in indoor-withering parameters (NML, 10.97%; NHS, 8.63%; *p* < 0.01). Terpenoids and esters are key volatile groups involved in the formation of the floral–fruity aroma of Oolong Tea, and changes in their relative contents and composition are closely related to aroma quality [32]. FHM showed the highest relative terpenoid content, with esters also remaining relatively abundant. This result agrees with the sensory evaluation, in which FHM showed the most prominent floral–fruity aroma and the best aroma quality.

Eighteen volatile organic compounds were shared by all six parameters (Figure 4C). These included seven terpenoids: D-limonene, linalool, (*E*)-farnesene, geranyl linalool, geraniol, pseudoionone, and (-)-isocedrol; three ketones: methyl heptenone, jasmone, and β-ionone; three esters: (*Z*)-3-hexenyl hexanoate, hexyl hexanoate, and delta-decalactone; two alcohols: dehydrolinalool and widdrol; and one aldehyde, one aromatic, and one phenol: benzaldehyde, o-cymene, and 2,4,6-tri-tert-butylphenol, respectively. As shown in Figure 4D, geraniol, β-ionone, and linalool were among the shared compounds with relatively high contents. Previous studies have shown that linalool and geraniol strongly influence tea flavor [33]. Linalool has a floral aroma and is an important volatile compound contributing to the floral–fruity aroma of Oolong Tea [34]. The relative content of linalool in teas produced using different processing techniques ranged from 1.74 to 3.83 µg/g, with the highest levels in FHM (3.83 µg/g) and RHL (2.25 µg/g) and the lowest level in RMM (1.74 µg/g). Geraniol has a rose-like aroma [35], and its relative content ranged from 2.52 to 7.41 µg/g, with the highest level in FHM (7.41 µg/g) and the lowest level in RMM (2.52 µg/g). β-ionone is also a floral compound and, together with related ionones, is produced by carotenoid degradation [36,37]. It is an important contributor to the floral–fruity aroma of Oolong Tea [38,39]. β-ionone was relatively high in RHL (3.41 µg/g) and lowest in NML (2.24 µg/g).

Linalool and geraniol have corresponding glycosidic precursors. These volatile compounds are mainly derived from the methylerythritol phosphate pathway [40]. Previous studies have reported that mechanical damage during shaking may influence β-glucosidase-related hydrolysis of glycosidic aroma precursors, which could contribute to the release of aroma compounds such as linalool and geraniol [41,42]. In the present study, the higher relative contents of linalool and geraniol in FHM may be associated with this process. Notably, the total relative content of shared volatile compounds was highest in FHM (42.2 µg/g). The high levels of linalool, which contributes sweet floral notes, and geraniol, which contributes rose-like notes, may act together to form the intense floral-fruity aroma of FHM. In contrast, NML likely showed weaker aroma because moderate shaking caused less glycoside hydrolysis. Volatile organic compounds in fresh tea leaves provide the basis for tea aroma [43], and many aroma compounds, including geraniol, linalool, and linalool oxides, occur as glycosides [44]. During tea processing, aroma precursors are converted into aroma compounds through reactions driven by light, heat, oxidation, and enzymatic activity [37]. Therefore, the higher relative content of shared volatile organic compounds in FHM suggests that this processing combination was associated with the accumulation of several key aroma-related volatiles. The underlying mechanisms, however, require further investigation.

Twenty-five parameter-specific volatile organic compounds were identified across the six parameters (Figure 4E). RMM contained three specific compounds: caryophyllene, 2-methylpentanoic anhydride, and 2,6-dimethyl-2,4,6-octatriene. RHL contained six specific compounds: Methyl hexanoate, methyl 2-hexenoate, 2,3,6-trimethyl-1,5-heptadiene, α-benzenepropanoic acid (hydroxyimino), 2-phenyloctane, and α-methylbenzyl acetate. NML contained two specific compounds: 3-pentadecanone and 3-methoxy-1-propanol. NHS contained three specific compounds: 1-ethenyl-3-methylenecyclopentene, isoamyl alcohol, and 2-cyclopentylidenecyclopentanone. FMS contained three specific compounds: 3-pyridin-3-ylbenzoic acid, o-Xylene, and β-myrcene. FHM contained eight specific compounds: ethyl 4-bromobenzoate, gamma-terpinene, 2,6,10-trimethyldodecane, 2-nonyne, decanal, cyclododecanone, α-ionone, and (*E*)-β-ionone. Among the specific compounds in RHL, short-chain aliphatic esters such as Methyl hexanoate and methyl 2-hexenoate generally have fruity characteristics [45,46]. FHM had the greatest number of specific volatile compounds (eight compounds). Among them, α-ionone and (*E*)-β-ionone are particularly important because both have low odor thresholds and strong floral notes, including violet-like and woody aromas [31], and are important aroma-active compounds in high-grade Oolong Tea [12,13]. These results suggest that FHM may promote the accumulation of specific terpenoids and ketones, thereby potentially shaping a richer and more complex aroma profile in Oolong Tea.

### 2.6. Screening of Key Volatile Organic Compounds

**Figure 5:**
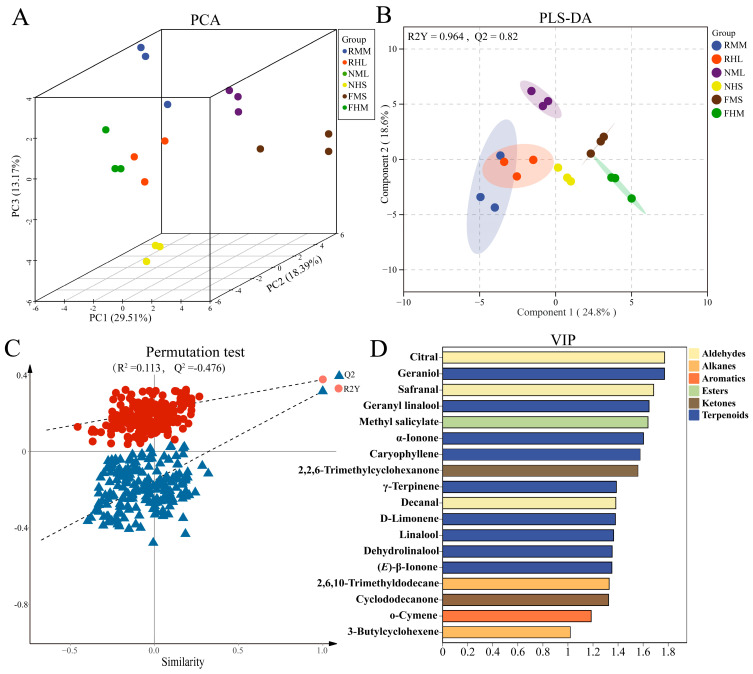
Structural analysis of volatile compounds in Oolong Tea under different processing parameters. (**A**) PCA; (**B**) PLS−DA; (**C**) permutation test of the PLS−DA model; (**D**) compounds with VIP > 1 based on the PLS−DA model.

Unsupervised PCA was used to evaluate volatile organic compounds in Oolong Tea produced using different processing techniques. The first three principal components explained 29.51%, 18.39%, and 13.17% of the aroma variation among processing parameters, respectively, and provided good separation between the parameters (Figure 5A). A PLS-DA model was then used to screen key components. As shown in Figure 5B, the model had a goodness-of-fit R^2^Y value of 0.964 and a predictive Q^2^ value of 0.82. The sun-withering groups (RMM and RHL) and combined-withering groups (FMS and FHM) each clustered relatively well, while different parameters remained clearly separated, indicating that processing techniques significantly affected the volatile organic compound composition of Oolong Tea. A 200-iteration permutation test (Figure 5C) showed that the intercept of the Q^2^ regression line with the vertical axis was less than 0, indicating no overfitting and confirming that the model was reliable (R^2^ = 0.113, Q^2^ = −0.476). The model was therefore used for screening key volatile organic compounds.

Using VIP > 1 and *p* < 0.05 as criteria, 18 key differential compounds were identified (Figure 5D). These included nine terpenoids: geraniol, geranyl linalool, α-ionone, γ-terpinene, D-limonene, linalool, dehydrolinalool, (*E*)-β-ionone, and caryophyllene; three aldehydes: citral, safranal, and decanal; two ketones: 2,2,6-trimethylcyclohexanone and cyclododecanone; two alkanes, 3-butylcyclohexene and 2,6,10-trimethyldodecane; one ester, methyl salicylate; and one aromatic compound, o-cymene. These compounds contributed strongly to the aroma composition of Oolong Tea produced using different processing techniques.

### 2.7. ROAV Analysis of Aroma Components Under Different Processing Techniques

The aroma characteristics of Oolong Tea arise from a highly complex volatile organic compound profile. However, the contribution of an individual compound to the overall aroma depends mainly on its odor threshold and relative content. Therefore, systematic screening and identification of key volatile compounds affecting Oolong Tea aroma are essential [47]. The ROAV is an important index for quantifying the contribution of volatile organic compounds to sample aroma. According to established criteria, compounds with ROAVs greater than 1 are generally considered to contribute significantly to aroma [48,49].

By calculating the ROAVs of key volatile organic compounds in Oolong Tea produced under different processing conditions, this study identified 22 key volatile organic compounds with an ROAV > 1 (Table 2). The numbers of compounds with an ROAV > 1 in RMM, RHL, NML, NHS, FMS and FHM were 13, 17, 16, 14, 15 and 16, respectively. Among them, geraniol (337.18–988.44), linalool (291.63–639.00), (*E*)-nerolidol (57.47–405.12), β-ionone (449.11–705.84), benzaldehyde (127.92–694.37), D-Limonene (71.49–147.76), Dehydrolinalool (9.40–43.88) and δ-Decalactone (17.85–147.60) shared key volatile organic compounds across all six parameters and made important contributions to the aroma characteristics of Oolong Tea. Because the ROAVs of individual compounds differed, teas produced by different processing techniques showed distinct aroma characteristics.

β-ionone is a low-threshold aroma compound that imparts a violet-like aroma and contributes substantially to Oolong Tea aroma formation. Benzaldehyde had a particularly high ROAV in NHS (ROAV = 694.37) compared with the other parameters. Shao et al. [50] reported that benzaldehyde has fruity and sweet notes, suggesting that benzaldehyde may be a major contributor to the evident floral–fruity aroma of NHS. Compared with the other parameters, (*E*)-nerolidol was particularly prominent in NML (ROAV = 405.12). Trans-nerolidol mainly contributes floral, citrus and woody notes [51]. In FHM, geraniol (ROAV = 988.44) and linalool (ROAV = 639.00) were particularly prominent and were the main sources of the strong floral aroma of this parameter. Linalool and geraniol can produce different aroma types at different ratios; when geraniol predominates, a rose-like aroma becomes more evident [52]. Therefore, the pronounced floral aroma and deep aroma persistence of FHM in this study may be mainly attributed to the high contribution of geraniol and linalool.

### 2.8. Correlation Analysis Between Quality-Related Compounds and Sensory Attributes

**Figure 6:**
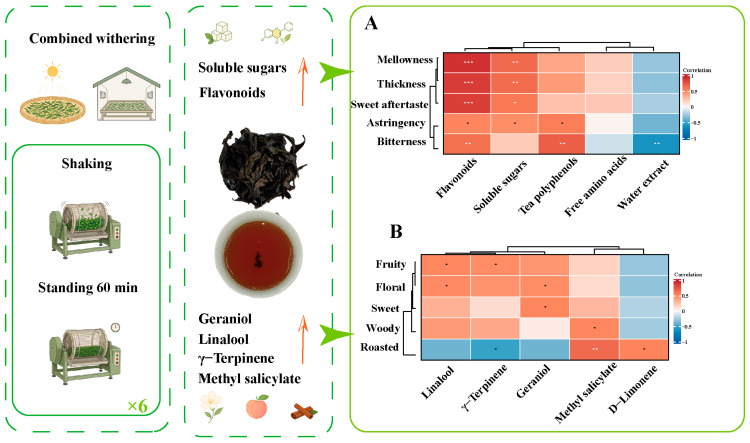
Correlation analysis between quality-related components and sensory attributes of HAOT. (**A**) Correlations between biochemical components and taste attributes. (**B**) Correlations between volatile organic compounds and aroma attributes. Red and blue colors indicate positive and negative correlations, respectively. *, ** and *** indicate significance at *p* < 0.05, *p* < 0.01 and *p* < 0.001, respectively. Orange arrows indicate increased accumulation of the corresponding metabolites under the optimized processing parameters.

To further clarify the chemical basis underlying flavor differences among HAOT samples, correlation analysis was performed between major quality-related compounds, aroma-active compounds and sensory attributes (Figure 6). Among the taste-related components, flavonoids showed relatively broad positive correlations with taste attributes. Specifically, flavonoid content was significantly positively correlated with mellowness, thickness and sweet aftertaste, indicating that, in the samples examined in this study, flavonoids were not only associated with bitterness and astringency but may also participate in the formation of mouthfeel fullness and taste complexity in tea infusions. Soluble sugars were also positively correlated with mellowness, thickness, and sweet aftertaste, suggesting that they may play an important role in enhancing sweetness perception and contributing to a mellow and full-bodied taste [27]. Tea polyphenols showed a different correlation pattern. Although tea polyphenols were positively correlated with some positive mouthfeel attributes, their correlations with bitterness and astringency were more evident, indicating that a relatively high tea polyphenol content may still be associated with harsh taste sensations [53]. In contrast, free amino acids showed relatively weak correlations with most taste attributes but tended to be negatively associated with bitterness, suggesting that free amino acids may partly alleviate bitter perception rather than directly determine the main taste differences among the processing parameters.

For aroma-related analysis, only aroma compounds that simultaneously met the criteria of VIP > 1, *p* < 0.05 and an ROAV > 1 were included, ensuring that the compounds discussed had both discriminatory ability among parameters and actual odor activity. The results showed that linalool was significantly positively correlated with fruity and floral attributes, whereas geraniol was significantly positively correlated with floral and sweet aroma attributes. Linalool is generally associated with floral and fruity notes, whereas geraniol mainly contributes rose-like floral notes [37]. Their simultaneous enrichment in FHM explained the intense and persistent floral–fruity aroma of FHM from both sensory and chemical perspectives. The correlation results also indicated that different aroma dimensions were associated with different types of aroma-active volatiles. γ-Terpinene was positively correlated with fruity aroma but negatively correlated with roasted aroma. In contrast, methyl salicylate was significantly positively correlated with woody aroma and showed the strongest positive correlation with roasted aroma. D-Limonene was also positively correlated with roasted aroma. The positive correlations of methyl salicylate and D-limonene with roasted aroma indicated that the roasted and woody sensory attributes in these samples were not solely determined by typical thermal reaction products, but may also be influenced by the co-variation of odor-active terpenoids and ester compounds [37,54].

## 3. Materials and Methods

### 3.1. Materials and Instruments

All experimental samples were collected from the tea gardens of Fujian Qilinshan Tea Industry Development Co., Ltd., in Minqing County, Fujian Province, China. The tea cultivar used was healthy ‘Rougui’, and fresh leaves were harvested in April 2025 at the stage of one bud with three to four leaves. High-altitude samples were obtained from tea gardens at 800–900 m above sea level (26.47° N, 118.96° E), which are typical high-mountain tea gardens in the region. The site has an annual average rainfall of approximately 1229 mm and an annual average relative humidity of approximately 75.5%. Additional tea samples from a low-altitude garden of the same company were used for paraffin sectioning.

The main instruments included a GCMS-QP2020 NX quadrupole gas chromatography–mass spectrometry system (Shimadzu, Kyoto, Japan), an SH-1-5SilMS capillary column (Shimadzu, Japan), a Waters 2695–2998 high-performance liquid chromatography system (Waters, Milford, MA, USA) and a 6CZQ-110 integrated tea green-making machine (Wuyishan Agang Tea Machinery Co., Ltd., Nanping, China).

### 3.2. Preparation of Paraffin Sections

Mature leaves (the first leaf) of ‘Rougui’ tea plants grown in high-altitude and low-altitude tea gardens were collected and cut into small pieces. The samples were fixed in a formalin-acetic acid-alcohol (FAA) fixative for 24 h. After fixation, the samples were dehydrated through a graded ethanol series (70%, 85%, 95% and 100%), cleared with xylene, infiltrated with paraffin and embedded in paraffin blocks. Sections were cut to a thickness of 8–10 μm using a rotary microtome. After drying, the sections were dewaxed with xylene and the residual xylene was removed through a graded ethanol series. The sections were then stained with safranin and fast green.

### 3.3. Orthogonal Experimental Design

Oolong Tea processing includes withering, green-making, fixation, rolling and drying. Previous studies have shown that the withering method (A), shaking intensity (B) and standing time (C) are key processing factors determining differences in tea quality [9,55,56]. Therefore, these three parameters were selected as experimental factors in a three-factor, three-level orthogonal design (Table S1).

### 3.4. Tea Processing

The processing conditions for each parameter were based on previous studies of Oolong Tea processing [14,24], with appropriate modifications. Withering parameters included solar withering, indoor withering and combined withering. Based on previous work by the research group [24], solar withering was conducted under a light intensity of 20,000 ± 1000 lx. Fresh leaves were spread evenly at a thickness of approximately 3 cm, with the ambient temperature maintained at 25 ± 2 °C and relative humidity at 55–65%. Leaves were gently turned every 10 min to avoid uneven withering until the moisture content decreased to 60%. Indoor withering was conducted under controlled temperature and humidity. Leaves were spread at approximately 5 cm thickness at 33 °C and 65% relative humidity and were turned every 30 min until the moisture content decreased to 60%. Combined withering consisted of 30 min of solar withering under the conditions described above (3 cm spreading thickness), followed by indoor withering at 33 °C and 65% relative humidity with a spreading thickness of 5 cm and turning every 30 min until the moisture content reached 60%.

Green-making mainly evaluated shaking intensity and standing time. Shaking intensity was divided into light, moderate and heavy levels. The leaf loading amount was controlled at approximately two-thirds of the capacity of the integrated green-making machine (40–45 kg). Light shaking included four rounds, with the first three conducted at 12 r/min for 1, 3 and 5 min respectively and the fourth conducted at 16 r/min for 3 min. Moderate shaking included five rounds, with the first three conducted at 12 r/min for 1, 3 and 5 min, respectively, and the last two conducted at 16 r/min for 3 and 8 min, respectively. Heavy shaking included six rounds, with the first three conducted at 12 r/min for 1, 3 and 5 min, respectively, and the last three conducted at 16 r/min for 3, 8 and 13 min, respectively. The standing time after shaking was selected as a processing factor because shaking and standing are performed alternately during the green-making process of Oolong Tea and the standing stage is closely related to water redistribution and subsequent metabolite transformation. Based on practical processing experience and the relevant literature [17], 60 min was selected as the moderate level, while 40 and 80 min were used to represent relatively short and long standing intervals, respectively.

All tea samples were fixed using a drum fixation machine at 260–280 °C for approximately 7 min. Fixed leaves were cooled to approximately 40–50 °C before rolling. Rolling was conducted for 6 min using a pressure sequence of light–moderate release–heavy release to curl the leaves tightly into strips. Drying was conducted using a three-stage baking and cooling procedure.

### 3.5. Sensory Evaluation

Sensory evaluation of the tea samples was conducted according to GB/T 23776-2018 (National Standard of the People’s Republic of China [57]). Briefly, the tea samples were first placed in a tea evaluation tray and their appearance was evaluated and scored. Subsequently, 5 g of each sample was placed in a standard evaluation cup and infused with 110 mL of boiling water. For the first infusion, the lid was opened after 1 min to evaluate the aroma, and the tea infusion was poured into the evaluation bowl after 2 min. The second and third infusions were then performed with steeping times of 3 min and 5 min, respectively. The evaluators scored the aroma, liquor color and taste based on the performance of the three infusions. After the infusions, the infused leaves were evaluated and scored. The total sensory score was calculated according to the scores and corresponding weights of appearance, liquor color, aroma, taste and infused leaves (Figure 2, Table S2). The descriptive terms and scoring criteria used for sensory evaluation were based on GB/T 14487-2017 [58].

QDA was further used to characterize the sensory attributes of HAOT samples (Figure S1). The evaluators were from Fujian Agriculture and Forestry University and were screened and trained for sensory evaluation. Their abilities to recognize, distinguish, describe and evaluate tea flavor attributes were assessed, with particular emphasis on their perception of the characteristic flavor profiles of HAOT. A total of 11 evaluators with a sensory identification accuracy of at least 80% were selected, including six females and five males aged 22–50 years. All evaluators were able to accurately use sensory descriptors and evaluate the samples using a 0–10 intensity scale, where 0 indicated the absence of an attribute and 10 indicated an extremely strong intensity. During evaluation, the evaluators recorded the taste and aroma characteristics of each sample. Finally, ten descriptors with a usage frequency of 80% or higher were selected to characterize the main sensory attributes of HAOT, including thickness, sweet aftertaste, astringency, mellowness, bitterness, roasted, floral, fruity, sweet and woody.

### 3.6. Determination of Conventional Macro-Compositions

Macro-compositions were measured in tea samples using different parameters. Water extract was determined according to GB/T 8305-2013 [59], Determination of Water Extracts in Tea. Tea polyphenols were determined according to GB/T 8313-2018 [60], Determination of Tea Polyphenols and Catechins in Tea. Free amino acids were determined according to GB/T 8314-2013 [61], Determination of Free Amino Acids in Tea. Flavonoid content was measured using the AlCl_3_ colorimetric method [62]. Soluble sugars were measured using a plant soluble sugar assay kit (Aladdin, Shanghai, China) according to the manufacturer’s instructions, with glucose used to establish the standard curve.

### 3.7. Determination of Volatile Organic Compounds

Volatile organic compounds were analyzed using HS-SPME-GC-MS. Tea powder (1 g, accurately weighed to 0.0001 g) was placed in a headspace vial, followed by the addition of 10 µL internal standard (ethyl decanoate, 10 µg/mL) and 5 mL boiling water. The vial was sealed, shaken evenly and equilibrated at 70 °C for 10 min on an electric heater. A 65 µm PDMS/DVB fiber (Supelco, Bellefonte, PA, USA) was then inserted for 40 min of adsorption before injection.

GC conditions were as follows: SH-1-5SilMS capillary column (30 m × 0.25 mm × 0.25 µm); helium carrier gas (purity > 99.999%) at 1.0 mL/min; injector temperature, 250 °C; ion source temperature, 230 °C; quadrupole temperature, 150 °C; transfer line temperature, 120 °C. The column temperature program was as follows: 50 °C for 1 min, then increased to 180 °C at 5 °C/min and held for 5 min, and finally increased to 230 °C at 10 °C/min and held for 8 min. MS conditions were as follows: electron ionization (EI) mode, ionization energy of 70 eV, scan range of 40–500 *m*/*z*, and solvent delay of 1 min. Each sample was analyzed in triplicate.

Retention indices (RI) were calculated using the linear retention indices of C7-C40 n-alkanes. RI values of volatile organic compounds were compared with reference values in the NIST Chemistry WebBook (https://webbook.nist.gov/chemistry, accessed on 23 June 2025) and with the literature data. Ethyl decanoate was used as the internal standard and the relative content of volatile organic compounds was calculated as follows:

$$Ai=\frac{Cis\times Mi}{Mis\times m}$$

where ‘*Ai*’ is the relative content of the volatile organic compound (µg/g), ‘*Mi*’ is the peak area of the compound, ‘*Mis*’ is the peak area of the internal standard, ‘*m*’ is the tea sample weight (g) and ‘*Cis*’ is the amount of ethyl decanoate (µg). The relative contents of volatile compounds were calculated using the internal standard method.

### 3.8. Data Processing

Each parameter was analyzed using three parallel replicates. Experimental data were organized in Excel 2007. PCA and PLS-DA were performed using SIMCA-P 14.1. Statistical analysis was performed using SPSS Statistics 27.0 software. Unless otherwise stated, other figures were produced using R software (v. 4.3). The relative odor activity value (*ROAV*) was used to compare the relative contribution of different volatile organic compounds to Oolong Tea aroma and was calculated as follows:

$$ROAVi=\frac{Ci}{Ti}$$

where ‘*Ci*’ is the relative content of the volatile organic compound in the sample and ‘*Ti*’ is the odor threshold of the compound in water.

## 4. Conclusions

This study systematically analyzed the effects of key processing parameters on the sensory quality, macro-compositions and volatile flavor characteristics of HAOT. Anatomical comparison showed that high-altitude tea leaves had thicker tissues and a more compact structure, which may influence water loss, cell damage and metabolite transformation during Oolong Tea processing. Range analysis of the orthogonal experiment showed that shaking intensity had the largest R value for sensory scores, followed by the withering method, whereas standing time showed a relatively small R value. Among the tested processing combinations, FHM, namely combined withering, heavy shaking and 60 min standing, showed the best overall quality. Therefore, in practical HAOT production, producers should give priority to optimizing shaking intensity, rather than focusing mainly on extending standing time, to better adapt the processing procedure to the structural characteristics of high-altitude fresh leaves.

FHM exhibited a persistent floral–fruity aroma, a mellow and full-bodied taste and a pronounced sweet aftertaste. Its higher flavonoid and soluble sugar contents may provide an important material basis for the formation of infusion thickness and sweetness. Volatile organic compound analysis showed that terpenoids and esters were the main classes of aroma compounds in HAOT. Multivariate statistical analysis further confirmed that different processing combinations formed clearly distinct volatile profiles and screened key differential volatiles, including geraniol, linalool, α-ionone, γ-terpinene, D-limonene and (*E*)-β-ionone. ROAV analysis further identified geraniol, linalool, β-ionone, (*E*)-nerolidol, benzaldehyde, D-Limonene, Dehydrolinalool and δ-Decalactone as important aroma-active compounds shared among different processing combinations. In FHM, the higher ROAVs and relative contents of geraniol and linalool provided a chemical explanation for its intense and persistent floral aroma.

Correlation analysis showed that flavonoids and soluble sugars were positively correlated with mellowness, thickness, and sweet aftertaste, while linalool and geraniol were closely associated with floral, fruity and sweet aroma attributes. Overall, FHM can be considered a promising processing parameter for HAOT under the present experimental conditions. Its quality improvement was mainly related to the coordinated accumulation of taste-related components and key aroma-active volatiles, providing a theoretical and practical basis for the targeted processing optimization of high-altitude Oolong Tea.

## Figures and Tables

**Table 1:** Orthogonal test results.

Run	Withering Method (A)	Shaking Intensity (B)	Standing Time (C)	Sensory Score
1	1 (solar)	1 (light)	1 (40 min)	73
2	1 (solar)	2 (moderate)	2 (60 min)	85
3	1 (solar)	3 (heavy)	3 (80 min)	89
4	2 (indoor)	1 (light)	2 (60 min)	71
5	2 (indoor)	2 (moderate)	3 (80 min)	83
6	2 (indoor)	3 (heavy)	1 (40 min)	88
7	3 (combined)	1 (light)	3 (80 min)	74
8	3 (combined)	2 (moderate)	1 (40 min)	86
9	3 (combined)	3 (heavy)	2 (60 min)	92
K1 (sum)	247	218	247	
K2 (sum)	242	254	248	
K3 (sum)	252	269	246	
k1 (mean)	82.33	72.67	82.33	
k2 (mean)	80.67	84.67	82.67	
k3 (mean)	84.00	89.67	82.00	
R (range)	3.33	17.00	0.67	
Factor order	B > A > C			
Favorable level	A3	B3	C2	
Favorable combination	A3B3C2 (FHM)			

**Table 2:** ROAVs of volatile compounds in Oolong Tea under different processing methods.

Volatile Organic Compounds	RT	RI	Odor Threshold (μg/g) [28,36]	ROAV						Odor Descriptor
				RMM	RHL	NML	NHS	FMS	FHM	
o-Xylene	8.02	870	0.450	0.00	0.00	0.00	0.00	2.87	0.00	Rose-like
Methyl hexanoate	9.938	928	0.07	0.00	6.73	0.00	0.00	0.00	0.00	Fruity
Isoamyl alcohol	10.14	934	0.004	0.00	0.00	76.30	134.82	0.00	0.00	Apple-like
Benzaldehyde	11.137	965	0.003	182.02	127.92	470.33	694.37	263.66	280.33	Fruity and sweet
Methyl heptenone	11.836	986	0.068	27.87	32.16	56.97	72.61	30.51	0.00	Fruity and fresh
D-Limonene	13.226	1029	0.034	147.46	74.43	78.05	117.26	121.30	71.49	Lemon-like
(*E*)-β-Ocimene	13.455	1036	0.034	12.30	38.48	0.00	31.23	30.85	0.00	Sweet and herbal
Phenylacetaldehyde	13.702	1044	0.004	0.00	75.17	236.40	0.00	0.00	124.66	Fruity
γ-Terpinene	14.128	1058	0.065	0.00	0.00	0.00	0.00	0.00	26.41	Herbal and woody
Linalool oxide	14.573	1072	0.19	6.15	13.11	10.70	13.37	0.00	11.00	Floral and woody
Linalool	15.46	1100	0.006	291.63	375.10	334.57	463.02	354.21	639.00	Floral and fruity
Dehydrolinalool	15.563	1102	0.11	31.37	9.40	43.88	19.79	12.43	22.51	Floral and woody
Methyl salicylate	18.945	1194	0.04	0.00	0.00	0.00	43.20	0.00	71.92	Wintergreen-like
Safranal	19.083	1197	0.003	0.00	266.76	219.63	0.00	296.64	0.00	Sweet
Geraniol	21.03	1249	0.007	337.18	550.91	432.69	804.26	713.18	988.44	Floral
Jasmone	26.07	1398	0.4	1.65	2.38	0.59	1.09	2.07	2.42	Jasmine-like
α-Ionone	27.111	1420	0.004	0.00	0.00	0.00	0.00	0.00	80.49	Violet-like and woody
α-Farnesene	29.802	1504	0.087	4.64	26.04	33.64	0.00	6.72	45.16	Citrus, herbal, and bergamot-like
(*E*)-Nerolidol	31.595	1563	0.01	394.50	293.11	405.12	57.47	369.38	150.49	Floral, citrus, and woody
Citral	32.087	1611	0.04	0.00	9.13	3.35	0.00	8.25	3.24	Sweet and lemon-like
β-Ionone	32.255	1617	0.005	449.11	680.96	449.22	705.84	575.85	579.37	Floral
δ-Decalactone	32.834	1635	0.031	65.16	71.43	17.85	96.46	44.59	147.60	Fruity and milky

RT represents retention time; RI represents retention index, namely the linear retention index calculated relative to a homologous series of n-alkanes.

## Data Availability

The original contributions presented in this study are included in the article/Supplementary Materials. Further inquiries can be directed to the corresponding author.

## Supplementary Materials

The following supporting information can be downloaded at https://www.mdpi.com/article/10.3390/plants15142125/s1. Table S1. Orthogonal experimental design for Oolong Tea processing. Table S2. Sensory evaluation of Oolong Tea produced using different processing methods. Figure S1. QDA scores of HAOT samples. (A) Aroma attributes, including roasted, floral, fruity, sweet, and woody aromas. (B) Taste attributes, including thickness, mellowness, sweet aftertaste, bitterness, and astringency.
