# Supplementary material for: Effects of Optimized Key Processing Parameters on Macro-Compositions and Volatile Organic Compounds in High-Altitude Oolong Tea (Camellia sinensis (L.) O. Kuntze)
Source: Plants (Basel). 2026 Jul 9;15(14):2125. doi: 10.3390/plants15142125 (PMC13414789; doi:10.3390/plants15142125)
Supplement: Supplementary file 1 [file plants-15-02125-s001.zip › plants-4402950-supplementary.pdf]

Table S1. Orthogonal experimental design for Oolong Tea processing.

Level	Withering method (A)	Shaking intensity (B)	Standing time (C)
1	Solar withering	Light	40 min
2	Indoor withering	Moderate	60 min
3	Combined withering	Heavy	80 min

Table S2. Sensory evaluation of Oolong Tea produced using different processing methods.

Sample	Appearance description (20%)	Liquor color description (5%)	Aroma description (30%)	Taste description (35%)	Infused leaves description (10%)	Total score
RMM	Relatively tight strips, dark and lustrous	Golden-yellow and bright	Moderately pronounced floral aroma	Mellow	Relatively soft and bright	85.00±2.75
RHL	Tight strips, greenish-brown and lustrous	Orange-yellow and bright	Pronounced floral aroma with a fruity note	Mellow, thick, and brisk	Plump, soft, and bright	89.01±1.61
NML	Relatively tight strips, dark and lustrous with a slight reddish tone	Orange-yellow and relatively bright	Fresh aroma	Mellow and slightly brisk	Relatively soft and bright	83.02±4.61
NHS	Tight strips, greenish-brown and lustrous	Orange-yellow and relatively bright	Floral-fruity aroma with a slight roasted note	Pure, mellow, and brisk	Plump, soft, and bright	88.04±4.10
FMS	Relatively tight strips, greenish-brown and lustrous	Orange-yellow and bright	Moderately pronounced floral-fruity aroma	Mellow and slightly brisk	Relatively soft and bright	85.95±3.94
FHM	Tight strips, greenish-brown and lustrous	Orange-yellow and bright	Intense and persistent floral-fruity aroma with a cinnamon-like note	Full-bodied with a sweet aftertaste	Plump, soft, and bright	92.00±2.24
RLS	Loose strips, less lustrous color	Golden-yellow liquor, slightly dull	Weak floral and fruity aromas with green note	Thick and fairly mellow	Slightly hard, greenish and dull	73.11±1.34
NLM	Loose strips, less lustrous color	Golden-yellow liquor, dull	Weak floral and fruity aromas with green note	Thick and fairly mellow, slightly coarse	Slightly hard, greenish and dull	71.00±3.43

FLL	Loose strips, less lustrous color	Golden-yellow liquor, slightly dull	Weak floral and fruity aromas with green note	Thick and fairly mellow	Slightly hard, greenish and dull	74.17±2.54

Figure S1. QDA scores of HAOT samples. (A) Aroma attributes, including roasted, floral, fruity, sweet, and woody aromas; (B) Taste attributes, including thickness, mellowness, sweet aftertaste, bitterness, and astringency.

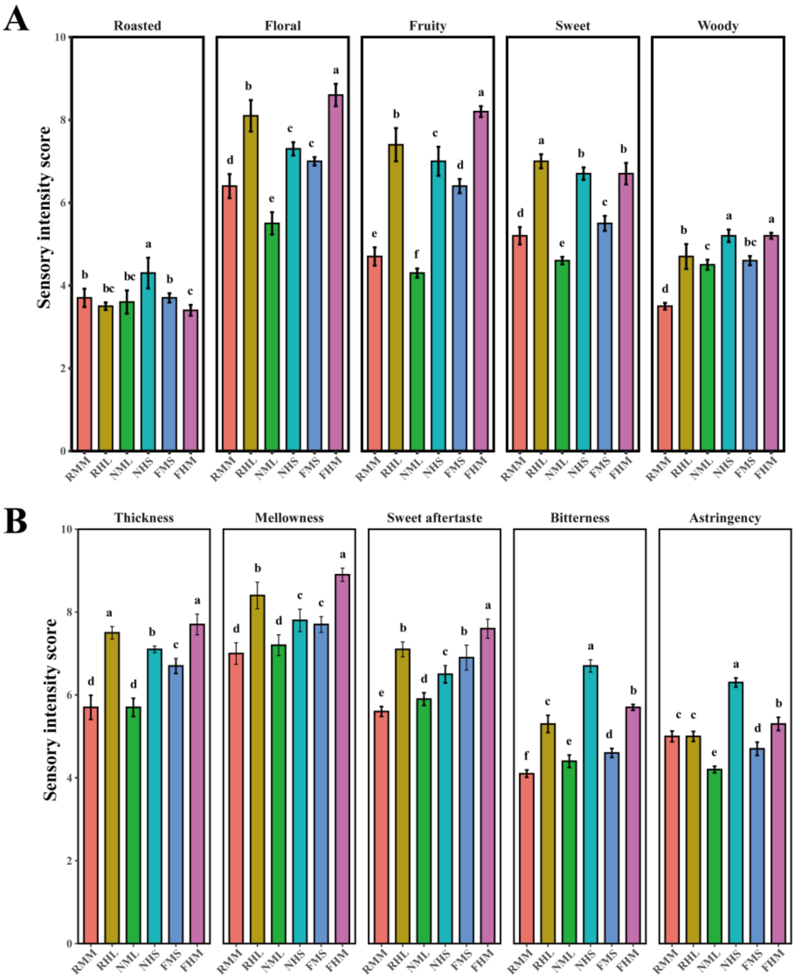

Data are expressed as mean ± SD. Different lowercase letters above the bars indicate significant differences among processing treatments within the same sensory attribute (one-way ANOVA followed by Tukey's HSD test, $p < 0.05$).
